# Biochemical Characterization of a Flavonoid *O*-methyltransferase from Perilla Leaves and Its Application in 7-Methoxyflavonoid Production

**DOI:** 10.3390/molecules25194455

**Published:** 2020-09-28

**Authors:** Hye Lin Park, Jae Chul Lee, Kyungha Lee, Jeong Min Lee, Hyo Jeong Nam, Seong Hee Bhoo, Tae Hoon Lee, Sang-Won Lee, Man-Ho Cho

**Affiliations:** 1 Department of Genetic Engineering, Kyung Hee University, Yongin 17104, Korea; hlpark@khu.ac.kr (H.L.P.); kyungha0714@khu.ac.kr (K.L.); mee7239@khu.ac.kr (J.M.L.); nhj4064@khu.ac.kr (H.J.N.); shbhoo@khu.ac.kr (S.H.B.); 2 Department of Applied Chemistry, Kyung Hee University, Yongin 17104, Korea; chemi1001@khu.ac.kr (J.C.L.); thlee@khu.ac.kr (T.H.L.); 3 Global Center for Pharmaceutical Ingredient Materials, Kyung Hee University, Yongin 17104, Korea

**Keywords:** PfOMT3, flavonoid 7-*O*-methyltransferase, perilla, 7-methoxyflavonoid, biotransformation

## Abstract

Methylation is a common structural modification that can alter and improve the biological activities of natural compounds. *O*-Methyltransferases (OMTs) catalyze the methylation of a wide array of secondary metabolites, including flavonoids, and are potentially useful tools for the biotechnological production of valuable natural products. An *OMT* gene (*PfOMT3*) was isolated from perilla leaves as a putative flavonoid OMT (FOMT). Phylogenetic analysis and sequence comparisons showed that PfOMT3 is a class II OMT. Recombinant PfOMT3 catalyzed the methylation of flavonoid substrates, whereas no methylated product was detected in PfOMT3 reactions with phenylpropanoid substrates. Structural analyses of the methylation products revealed that PfOMT3 regiospecifically transfers a methyl group to the 7-OH of flavonoids. These results indicate that PfOMT3 is an FOMT that catalyzes the 7-*O*-methylation of flavonoids. PfOMT3 methylated diverse flavonoids regardless of their backbone structure. Chrysin, naringenin and apigenin were found to be the preferred substrates of PfOMT3. Recombinant PfOMT3 showed moderate OMT activity toward eriodictyol, luteolin and kaempferol. To assess the biotechnological potential of PfOMT3, the biotransformation of flavonoids was performed using *PfOMT3*-transformed *Escherichia coli*. Naringenin and kaempferol were successfully bioconverted to the 7-methylated products sakuranetin and rhamnocitrin, respectively, by *E. coli* harboring *PfOMT3*.

## 1. Introduction

Flavonoids are a structurally diverse group of plant secondary metabolites, which play important roles in plant growth and defense against biotic and abiotic stresses [1,2,3]. As phytonutrients in the human diet, they have a wide range of health-beneficial properties including antioxidant, antimicrobial, anti-inflammatory and anticancer activities [3,4]. Flavonoids are divided into several subclasses, such as flavanones, flavones, flavonols, isoflavones and anthocyanins, according to the oxidation states and specific substitutions on their pyran ring [4,5]. In addition, a wide range of modifications of the hydroxyl groups, such as glycosylation, methylation and prenylation, offer structural diversity to flavonoids, and affect their physiological functions and biological activities [1,4]. *O*-Methyltransferases (OMTs) is a common modification found in flavonoids, and is catalyzed by *S*-adenosyl-L-methionine (SAM)-dependent OMTs [6,7,8,9].

The methylation of flavonoids can alter and/or improve their physicochemical properties and biological activities [6,7,8,9]. Methylated chrysin and apigenin showed improved hepatic metabolic stability and intestinal absorption [10]. The methylation of genistein and kaempferol enhanced their affinity for proteins [11]. Thus, methylation has been suggested to be a simple and effective way to enhance the metabolic resistance and transport of ingested flavonoids [9,11]. Methylation is important in the antimicrobial activity of flavonoids. Ponciretin (4′-*O*-methylnaringenin) and sakuranetin (7-O-methylnaringenin) were shown to have antibacterial activity against *Helicobacter pylori* [12]. Sakuranetin is a rice phytoalexin against fungal and bacterial phytopathogens [13,14]. 7-*O*-methylation of naringenin was shown to enhance antimicrobial activity against the rice blast pathogen [13].

Biotechnological approaches for modifying natural products have been attempted in order to develop and produce valuable compounds [5,15,16,17]. Many flavonoid OMTs (FOMTs) transfer methyl groups onto specific hydroxyl groups of flavonoids [18,19,20,21]. FOMTs commonly accept a variety of flavonoids as substrates with different catalytic efficiencies [5,20,21,22]. Therefore, FOMTs are a useful tool for the biotechnological production of methoxyflavonoids. Several studies have demonstrated that the biotransformation of flavonoids using engineered microorganisms bearing an FOMT gene is a promising method for the production of desired methoxyflavonoids [18,20,22].

Perilla (*Perilla frutescens*) is a spicy vegetable widely cultivated in Asian countries [23,24,25]. The leaves and seeds of perilla have long been used as traditional Chinese medicines to treat various conditions, such as the common cold, headaches and coughing [23,24,25]. Perilla leaves produce a wide array of phenolics and terpenes [23,24,25,26]. Volatile compounds in perilla leaves are mainly monoterpenes (perillaldehydes and perillaketones) and phenylpropenes (myristicin, elemicin and dillapiole) [23,25,26]. The hydrophilic secondary metabolites in perilla leaves include rosmarinic acid and its derivatives, and flavonoids including apigenin, luteolin and chrysoeriol [24,25]. The hydroxyl groups of many of the phenolic compounds found in perilla are highly methylated. Myristicin, elemicin and dillapiole are methylated forms of phenylpropenes, and chrysoeriol is a methoxyflavonoid.

The richness of methylated phenolic compounds in perilla leaves suggested that perilla may be a good genetic resource for OMTs. In the present study, we isolated and identified an OMT gene encoding an FOMT from perilla leaves. Phylogenetic analysis showed that the isolated OMT is a class II OMT. A recombinant OMT protein catalyzed the methylation of flavonoid substrates. Structural analyses of the methylation products showed that the isolated OMT regio-specifically transfers a methyl group to the 7-OH of flavonoids. For the biotechnological applications of the identified OMT, the biotransformation of flavonoids using *E. coli* harboring the *OMT* gene was performed to produce 7-methoxyfavonoids.

## 2. Results and Discussion

### 2.1. Cloning and Molecular Characterization of PfOMT3 from Perilla Leaves

**Figure 1:**
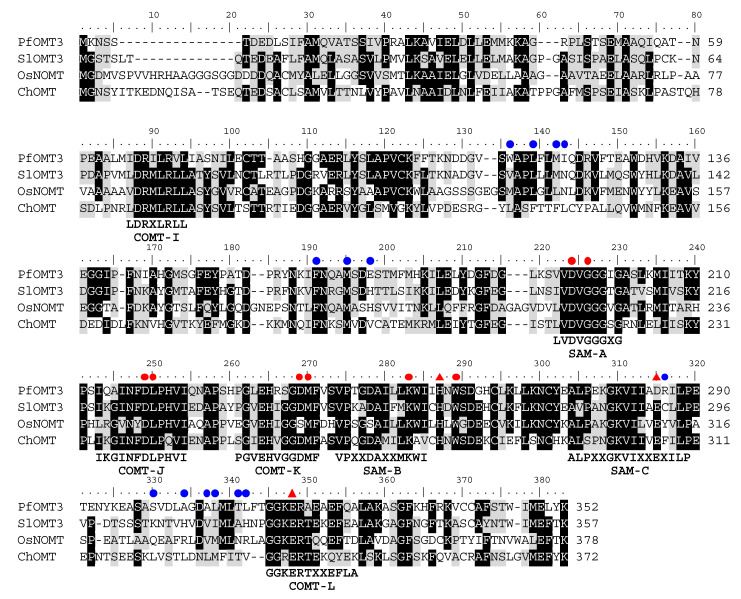
Multiple alignment of *Perilla frutescens O*-methyltransferase3 (PfOMT3) amino acid sequence with other class II *O*-methyltransferases (OMTs). Amino acid sequences of *Solanum lycopercicum* OMT3 (SlOMT3), *Oryza sativa* naringenin OMT (OsNOMT) and chalcone OMT (ChOMT) from alfalfa were used in the alignment. Three *S*-adenosyl-L-methionine (SAM)-binding motifs (SAM-A, B and C) and four caffeic acid OMT (COMT) motifs (COMT-I, J, K and L) conserved in class II OMTs are indicated with their consensus sequences under the aligned sequences. Red triangles indicate the three catalytic residues of class II OMTs. Red and blue circles indicate SAM-binding residues and substrate-binding residues, respectively. Identical and similar residues are shaded in black and gray, respectively.

Based on the perilla transcriptome [27], we designed primer sets to clone *FOMT* genes from perilla leaves. Several OMT genes were amplified from perilla leaves. Among them, an *OMT* gene was identified as a putative FOMT, and named *P. frutescens OMT3* (*PfOMT3*, GenBank accession number: MT909556). This gene has an open leading frame of 1059 base pairs that encodes a 352-amino acid polypeptide chain (Figure 1 and Figure S1). Analysis of protein families with the search tool in Pfam (https://pfam.xfam.org) and PROSITE (https://prosite.expasy.org/) revealed that PfOMT3 is a class II SAM-dependent OMT containing a methyltransferase 2 domain and a dimerization domain. SAM-dependent OMTs participate in the methylation of a wide array of secondary metabolites, which are divided into two major classes [7,8,28]. Class I OMTs are Mg^2+^-dependent, small (molecular mass of 26–30 kDa) OMTs, such as caffeoyl-coenzyme A OMTs (CCoAOMTs) [7,8,28]. Class II OMTs include higher molecular mass OMTs (36–43 kDa) that catalyze the methylation of diverse secondary metabolites [7,8,28]. The theoretical molecular mass of PfOMT3 is 38.8 kDa, which is consistent with it being a class II OMT.

A multiple sequence alignment of PfOMT3 and other plant OMTs revealed that PfOMT3 has the signature motifs of class II OMTs (Figure 1). Three SAM-binding motifs (SAM-A, B and C) are conserved between both class I and class II OMTs [28]. These motifs are located on the C-terminus of class II OMTs, including PfOMT3 (Figure 1). In addition to SAM-binding motifs, Caffeic acid OMT (COMT) motifs (COMT-I, J, K, and L) are present in class II OMTs [7,28]. Caffeic acid OMT (COMT) is a representative class II OMT, and catalyzes the methylation of hydroxycinnamic acids, including caffeic acid and 5-hydroxyferulic acid, thereby participating in lignin biosynthesis [7,8]. PfOMT3 had four highly conserved COMT motifs (Figure 1).

The catalytic residues (His, Asp/Glu and Glu), SAM-binding residues and substrate-binding residues of class II OMTs were identified by structural and sequence comparisons of ChOMT and IOMT with other OMTs [29]. Although class II OMTs commonly use SAM as a methyl donor, they differ in their selectivity to classes of secondary metabolites such as hydroxycinnamic acids, flavonoids, coumarins and alkaloids [7,28]. Three catalytic residues (His257, Asp285 and Glu318) and SAM-binding residues were highly conserved between PfOMT3 and other class II OMTs (Figure 1). However, substrate-binding residues were variable among class II OMTs, likely reflecting their different substrate specificity (Figure 1) [29].

**Figure 2:**
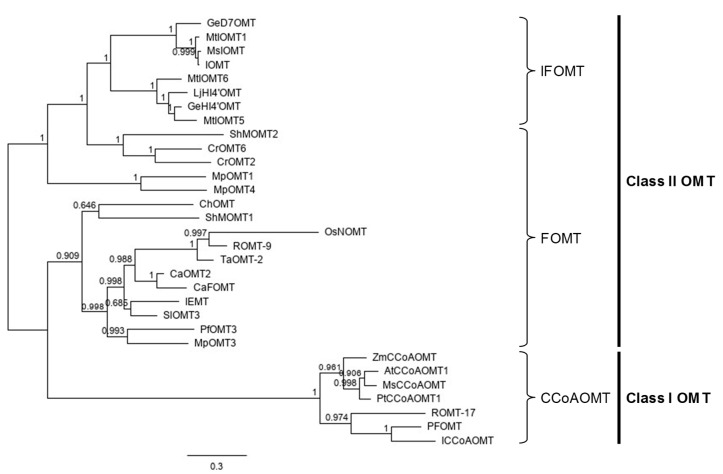
Phylogenetic tree of PfOMT3 and other plant OMTs. Phylogenetic analysis was conducted with the Bayesian method using MrBayes program. Class I OMTs (CCoAOMTs) were clearly separated from class II OMTs. Class II OMTs include flavonoid OMTs (FOMTs) and isoflavonoid OMTs (IFOMTs) according to their preferred substrate class. Numbers above branches represent the posterior probabilities.

Phylogenetic analysis of SAM-dependent OMTs and PfOMT3 was performed (Figure 2). Class II OMTs were clearly separated from class I OMTs. Consistent with the results of protein family analysis, phylogenetic analysis showed that PfOMT3 is a class II OMT. Class II OMTs include FOMTs and isoflavonoid OMTs (IFOMTs). PfOMT3 grouped together with the FOMTs. (Figure 2).

### 2.2. PfOMT3 is a Flavonoid 7-O-Methyltransferase

**Figure 3:**
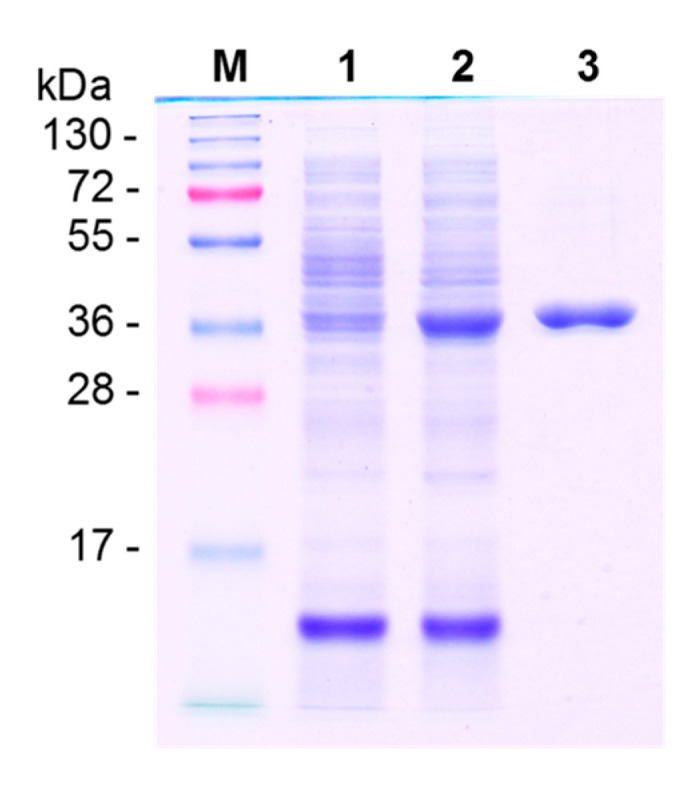
Expression and purification of recombinant PfOMT3. M, size marker; 1, crude extract before isopropyl β-D-1-thiogalactopyranoside (IPTG)-induction; 2, crude extract after IPTG-induction; 3, Ni^2+^-affinity purified PfOMT3.

To elucidate the biochemical properties, including substrate selectivity, regiospecificity and catalytic efficiency of PfOMT3, recombinant PfOMT3 was produced in *E. coli*. *PfOMT3* was inserted into the pET28a(+) vector for expression of the PfOMT3 protein as a His-tag fusion protein. PfOMT3 was successfully expressed in the *E. coli* BL21 cell (Figure 3). Recombinant PfOMT3 was purified by Ni^2+^-affinity chromatography to apparent homogeneity (Figure 3). The molecular mass of the purified PfOMT3 as determined by SDS-PAGE was 40.96 kDa, which is in good agreement with the theoretical molecular mass of His-tagged PfOMT3.

**Figure 4:**
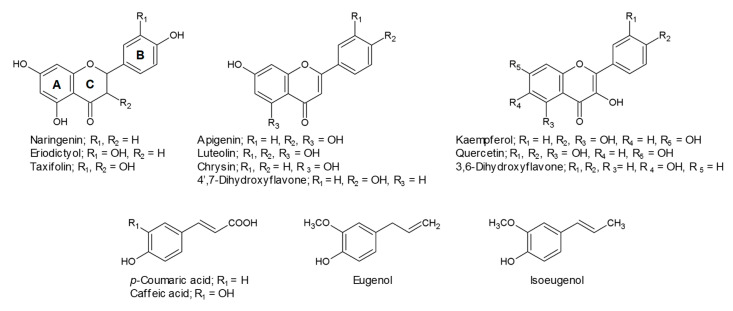
Structures of flavonoid and phenylpropanoid substrates used in the PfOMT3 activity assay. The letters indicate the lettering system for the three flavonoid rings.

**Figure 5:**
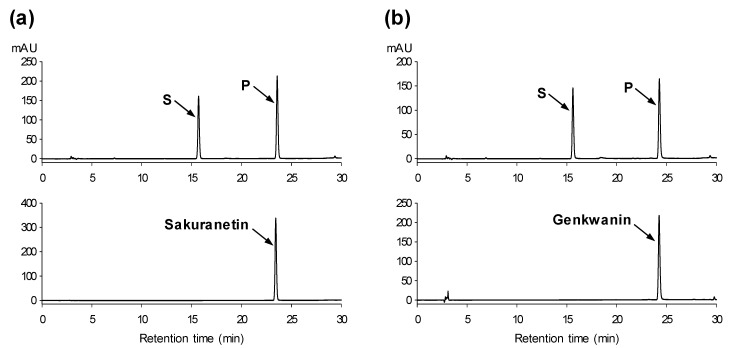
Representative high-performance liquid chromatography (HPLC) chromatograms of PfOMT3 reactions with flavonoid substrates. Naringenin (**a**) and apigenin (**b**) were converted to their corresponding methylated products by recombinant PfOMT3. S, substrate; P, methylated product. Upper panel: HPLC chromatograms of the PfOMT3 reaction mixtures. Lower panel: HPLC chromatograms of the authentic 7-*O*-methylated flavonoids, sakuranetin and genkwanin.

The OMT activity of recombinant PfOMT3 was assayed with flavonoids and phenylpropanoids (hydroxycinnamic acids and phenylpropenes) to determine substrate selectivity. Flavonoid substrates with different backbones, flavanones (naringenin, eriodictyol and taxifolin), flavones (apigenin, luteolin, chrysin and 4′,7-dihydroxyflavone) and flavonols (kaempferol, quercetin and 3,6-dihydroxyflavone) were used for the PfOMT3 activity assay (Figure 4). Two hydroxycinnamic acids, *p*-coumaric acid and caffeic acid, were examined as phenylpropanoid substrates. Perilla leaves contain diverse methylated phenylpropenes that are methylated derivatives of eugenol [23,25,26]. The methylation of phenylpropenes has been reported to be catalyzed by class II OMTs [30,31]. We therefore investigated if eugenol and isoeugenol, as precursors of methylated phenylpropenes, were substrates for PfOMT3. Phylogenetic analysis suggested that PfOMT3 is an FOMT (Figure 2). Consistent with this finding, the OMT assays showed that recombinant PfOMT3 accepted most flavonoid substrates and yielded methylated products (Figure 5 and Figure S2). No product peaks were detected in PfOMT3 reactions with phenylpropanoid substrates (Figure S3). These findings suggest that PfOMT3 selectively uses flavonoids as methyl acceptor molecules.

In addition to substrate selectivity, many class II OMTs have been shown to transfer methyl groups to specific phenolic hydroxyl groups [5,18,19,20,21,22,32,33]. A set of regiospecific OMTs was reported to be involved in the biosynthesis of polymethoxyflavonoids with different methylation patterns in sweet basil [32]. In our activity assays, PfOMT3 catalyzed the methylation of most examined flavonoids regardless of their backbones (Figure S2 and Table 1). Two flavonoids with no OH groups in the B ring, namely chrysin and 3,6-dihydroxyflavone, were used in the OMT assay to determine possible hydroxyl groups that are methylated by PfOMT3. Chrysin was methylated by PfOMT3, whereas 3,6-dihydroxyflavone was not (Figure S2). The methylation of chrysin indicates that the OH groups in the B ring are not required for the PfOMT3 methylation. The inability of PfOMT3 to methylate 3,6-dihydroxyflavone also suggested that PfOMT3 does not transfer a methyl group to the 3- and 6-OH groups of the A ring. All flavonoid substrates used by PfOMT3 as methyl acceptors in our experiments contain both 5- and 7-OH groups, suggesting that these two OH groups are possible positions of PfOMT3 methylation. Methylated products of naringenin and apigenin showed the same retention times as sakuranetin and genkwanin (7-*O*-methylapigenin), respectively, suggesting that PfOMT3 likely transfers a methyl group to the 7-OH of flavonoid substrates (Figure 5).

To confirm the regiospecificity of the PfOMT3 reaction, methylated products of naringenin and kaempferol were analyzed by NMR spectroscopy. In the ^1^H NMR spectra, a single methoxy proton signal was observed in the methylated product of both naringenin and kaempferol, indicating the mono-methylation of flavonoid substrates by PfOMT3. Methoxy proton signals in methylated-naringenin and methylated-kaempferol were observed at δ 3.80 and δ 3.88, respectively (Table S1). Methoxy carbon signals were observed at δ 54.85 and δ 55.02 in the methylated products of naringenin and kaempferol, respectively (Table S1). Because HPLC analysis suggested the 7-*O*-methylation of flavonoids by PfOMT3 (Figure 5), the correlations between methoxy proton signals and C-7 signals in the methylated products were analyzed by heteronuclear multiple bond correlation (HMBC) spectroscopy. In the ^13^C NMR spectra, C-7 signals were observed at δ 168.14 and δ 165.66 in the methylated-naringenin and methylated-kaempferol, respectively (Table S1). The methoxy proton signals in both products were correlated with the C-7 signals in HMBC spectra, confirming that the methylation products of naringenin and kaempferol are sakuranetin and rhamnocitrin (7-*O*-methylkaempferol), respectively. The NMR results for the methylated products together with the HPLC results for the PfOMT3 reactions indicated that PfOMT3 catalyzes the 7-*O*-methylation of flavonoids.

### 2.3. Substrate Preference of PfOMT3

Class II OMTs use diverse compounds within the same class of secondary metabolites as methyl accepter molecules [5,7,8]. Many studies have also shown that FOMTs accept a variety of flavonoids as substrates with different preferences [18,19,20,21,22,32,33]. OsNOMT, a flavonoid 7-OMT from rice, methylated diverse flavonoids such as naringenin, kaempferol, apigenin, luteolin, liquiritigenin and quercetin, with the highest preference for the flavanone naringenin [21]. However, it showed almost no activity toward myricetin [21]. The 3′/5′-OMT SlOMT3 from tomato preferably methylated the flavonols quercetin and laricitrin, while it showed less activity when provided with the flavanone eriodictyol or the flavone luteolin as a substrate [20]. Although Pa4′OMT from liverwort was identified as an apigenin 4′-OMT, it also catalyzed the methylation of other flavonoids such as luteolin, scutellarein, genkwanin, quercetin and kaempferol [33].

PfOMT3 methylated diverse flavonoids with different activities (Table 1). Under our experimental conditions (see Methods), PfOMT3 showed the highest OMT activity toward chrysin. The activity of PfOMT3 towards naringenin and apigenin was 97.79% and 95.17%, relative to its activity towards chrysin, respectively, suggesting that chrysin, naringenin and apigenin are the preferred substrates of PfOMT3. Recombinant PfOMT3 showed moderate activities toward eriodictyol, luteolin, 4′,7-dihydroxyflavone and kaempferol, with activities of 53.13%, 46.80%, 62.76% and 40.34%, respectively, relative to chrysin (Table 1). Although taxifolin and quercetin contain a 7-OH group, 7-*O*-methylated products were not detected in PfOMT3 reactions with these substrates (Table 1 and Figure S2).

The kinetic properties of PfOMT3’s reaction to selected flavonoid substrates were examined (Table 2). PfOMT3 showed the highest affinity for chrysin, with a *K*_M_ value of 1.31 μM, among the flavonoids examined. PfOMT3 had a lower *K*_M_ value for apigenin. The *K*_M_ values of PfOMT3 for naringenin, eriodictyol and kaempferol were 13.4 μM, 13.08 μM and 17.88 μM, respectively. The highest *K*_M_ value was obtained for luteolin. The *K*_M_ values of PfOMT3 toward flavonoids are comparable to those of other FOMTs [20,21,22,33,34]. Although PfOMT3 only had moderate affinity for naringenin, it had the highest *V*_max_ value for this substrate. Therefore, PfOMT3 had the highest catalytic efficiency for naringenin, with a *k*_cat_/*K*_M_ value of 2.632 × 10^3^ M^−1^ s^−1^. The *k*_cat_/*K*_M_ values of apigenin and chrysin were comparable to that of naringenin. Luteolin showed the lowest *k*_cat_/*K*_M_ value among the examined flavonoids. Similar to the results obtained from relative activity assays of PfOMT3, kinetic analysis demonstrated that naringenin, chrysin and apigenin are preferred substrates of PfOMT3.

### 2.4. Application of PfOMT3 in the Biotechnological Production of 7-Methoxyflavonoids

**Figure 6:**
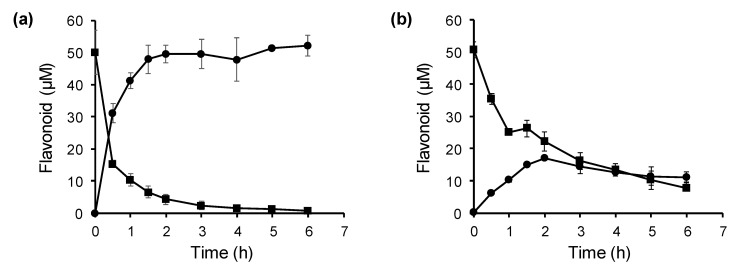
Kinetics of biotransformation of flavonoid substrates (■) to the corresponding methylated products (●) using *E. coli* harboring *PfOMT3*. Bioconversions of (**a**) naringenin to sakuranetin, and (**b**) kaempferol to rhamnocitrin, were monitored over time. Results are the means ± SD of three independent experiments.

FOMTs have potential applications in the biotechnological production of methoxyflavonoids [5,20,34,35,36]. In the present study, *E. coli* was engineered to harbor the *PfOMT3* gene and then used to produce 7-methoxyflavonoids from flavonoids. After the induction of PfOMT3, the *E. coli* culture was transferred into fresh medium followed by the addition of either naringenin or kaempferol at 50 μM. As a preferred substrate of PfOMT3, naringenin was quickly bioconverted by the engineered *E. coli* to sakuranetin compared with kaempferol (Figure 6a). Most naringenin was consumed within 3 h and an equivalent amount of sakuranetin was produced. Kaempferol was converted to rhamnocitrin in the *E. coli* culture at a slower rate than naringenin (Figure 6b). This result is consistent with the lower activity of PfOMT3 in relation to kaempferol than in relation to naringenin. The rhamnocitrin content reached a maximum level 2 h after biotransformation, and then decreased slightly (Figure 6b). The maximum yield of rhamnocitrin produced by the engineered *E. coli* culture was 33.9%. Although production rates and yields were different, sakuranetin and rhamnocitrin were produced from non-methylated precursors by biotransformation using the engineered *E. coli*. This result indicates that the microorganisms bearing *PfOMT3* can be used for the biotechnological production of 7-methoxyflavonoids.

Given their substrate-, regio- and stereo-selectivities, biosynthetic enzymes are considered useful tools for the modification of synthetic and natural compounds [15,16]. Thus, genetically engineered microorganisms transformed with biosynthetic genes, including *FOMT*s, have been used for the biotechnological production of valuable secondary metabolites [5,15,16,17]. The bioconversion yields of flavonoids to methoxyflavonoids using engineered microorganisms differ according to the FOMTs and substrates used, substrate concentrations, and culture conditions [5,22,34,35,36]. *E. coli* containing *SlOMT3* efficiently converted luteolin and eriodictyol to chrysoeriol and homoeriodictyol, respectively, with more than 90% yields at a substrate concentration of 50 μM [20]. Under the same conditions, the maximum yield of quercetin to isorhamnetin by *SlOMT3*-transformed *E. coli* was 59.8% [20]. Similarly, most supplied naringenin was converted to sakuranetin by our *PfOMT3*-transformed *E. coli* culture, while the bioconversion yield of kaempferol to rhamnocitrin was much lower than that of naringenin (Figure 6). Several studies have demonstrated that the bioconversion efficiency of flavonoids using engineered microorganisms can be improved by the optimization of biotransformation conditions. The bioconversion yields of genistein to methylated genistein by *E. coli* bearing *SaOMT2* from *Streptomyces avermitilis* differed widely according to substrate concentration, with 65% and 3% yields at substrate concentrations of 100 and 1000 μM, respectively [36]. Changing the culture medium was shown to increase the methylation yield of 7,8-dihydroxyflavone by *E. coli* expressing *SpOMT2884* [32]. The enhanced internal production of SAM resulted in the increased production of methoxyisoflavonoids by *E. coli* bearing *SaOMT2* and *metK* encoding SAM synthase [36]. To increase the bioconversion efficiency of *PfOMT3*-transformed *E. coli* with less preferred substrates, including kaempferol, the biotransformation conditions will need to be further optimized.

## 3. Materials and Methods

### 3.1. Materials

Perilla leaves were purchased from a local market in Korea. The flavonoids used were obtained from Indofine Chemical Company (Hillsborough, NJ, USA) and Extrasynthese (Genay Cedex, France). *p*-Coumaric acid, eugenol and isoeugenol were purchased from Sigma-Aldrich (St. Louis, MO, USA). Caffeic acid was obtained from Indofine Chemical Company. SAM was purchased from Sigma-Aldrich. HPLC grade solvents and other reagents were obtained from Sigma-Aldrich, Duchefa Biochemie (Haarlem, The Netherlands), and Samchun Chemicals (Seoul, Korea).

### 3.2. Cloning and Phylogenetic Analysis of PfOMT3

First strand cDNA was synthesized from total RNA isolated from perilla leaves using SuPrimeScript RT premix (GeNet Bio, Daejeon, Korea) with an oligo dT primer. *PfOMT3* was amplified from the first strand cDNA using Solg^TM^ Pfu DNA Polymerase (SolGent, Daejeon, Korea). The primer set used to amplify *PfOMT3* was GGCATATGAAGAATTCATCAACGGATGA and CCGTCGACTTATTTGTAGAGTTCCATGATCC. The resulting PCR product was subcloned into the pJET 1.2 blunt cloning vector (Thermo-Fisher Scientific, Waltham, MA, USA). After confirming the sequence, *PfOMT3* was inserted into the pET28a(+) vector (Novagen, Madison, WI, USA). The resulting *PfOMT3*/pET28a(+) construct was transformed into *E. coli* BL21(DE3) cells.

The multiple alignment of amino acid sequences of SAM-dependent OMTs was conducted with Clustal Omega [37]. Phylogenetic analysis was carried out with the Bayesian method using MrBayes program [38]. The Markov chain Monte Carlo settings for the phylogenetic analysis were four independent runs for 1,000,000 generations sampled every 1000 generations and burn-in length of 100,000. The OMT sequences used to generate the phylogenetic tree were ShMOMT1 (JF499656), ShMOMT2 (JF499657), ChOMT (AAB48059), MpOMT1 (AY337457), MpOMT3 (AY337460), MpOMT4 (AY337461), ROMT-9 (DQ288259), TaOMT-2 (DQ223971), OsNOMT (AB692949), CaOMT2 (U16793), CaFOMT (U16794), SlOMT3 (AK325603), IEMT (U86760), CrOMT2 (AY127568), CrOMT6 (AY343489), GeHI4OMT (AB091684), MtIOMT6 (DQ419913), MtIOMT5 (AY942158), MtIOMT1 (AY942159), LjHI4OMT (AB091686), GeD7OMT (AB091685), MsIOMT (U97125), IOMT (AAC49927), PFOMT (AY145521), ICCoAOMT (L22203), ROMT-17 (XM_507282), MsCCoAOMT (U20736), AtCCoAOMT1 (AY057554), PtCCoAOMT1 (AJ224894) and ZmCCoAOMT (AJ242980).

### 3.3. Heterologous Expression and Purification of Recombinant PfOMT3

*E. coli* cells bearing the *PfOMT3*/pET28a(+) construct were grown at 37 °C in LB medium containing kanamycin (25 µg/mL) until an OD_600_ of ~0.6 was reached, at which time 0.1 mM isopropyl β-D-1-thiogalactopyranoside (IPTG) was added to the culture to induce the production of PfOMT3. Cells were further grown for 16 h at 25 °C. After induction, cells were harvested by centrifugation (5000× *g* for 15 min) and the resulting cell pellets were resuspended in phosphate-buffered saline (137 mM NaCl, 2.7 mM KCl, 10 mM Na_2_HPO_4_, 2 mM KH_2_PO_4_) supplemented with lysozyme (1 mg/mL) and phenylmethylsulfonyl fluoride (1 mM). Cells were lysed by sonication on ice, and then centrifuged at 15,900× *g* for 20 min at 4 °C. Recombinant PfOMT3 was purified from the crude extract by affinity chromatography using Ni-NTA agarose beads (Qiagen, Hilden, Germany). PfOMT3 was eluted with 100 to 150 mM imidazole in Tris buffer (50 mM Tris, pH 8.0, 300 mM NaCl). Eluted proteins were analyzed by sodium dodecyl sulfate-polyacrylamide gel electrophoresis.

### 3.4. OMT Activity Assay and Kinetic Analysis

OMT activity was assayed in a reaction mixture with a final volume of 500 µL containing either flavonoid or phenylpropanoid substrate (50 µM), SAM (100 µM) and recombinant PfOMT3 (50 μg) in Tris-HCl buffer (20 mM, pH 7.5). After a 30 min incubation at 37 °C, reactions were stopped by adding 5 M HCl (50 μL). Reaction products were extracted with ethyl acetate twice, and the extracts were then evaporated to dryness. The residues were redissolved in methanol and subjected to HPLC analysis. After stopping the reaction, reaction mixtures with phenylpropanoid substrates were subjected directly to HPLC analysis. Reaction products were resolved by HPLC using a Sunfire C18 column (Waters, Milford, MA, USA). Elution was performed with a linear gradient of 25–60% acetonitrile in 3% acetic acid-water for 25 min at a flow rate of 1 mL/min with detection at 280 nm. Kinetic analysis of PfOMT3 reactions was performed by varying the concentrations (0.5–100 μM) of flavonoid substrates. Kinetic assays were performed in triplicate and results are presented as means ± standard deviations.

### 3.5. Biotransformation of Flavonoids using E. coli Bearing PfOMT3

Engineered *E. coli* harboring *PfOMT3* were grown and induced with IPTG as described above. After a 4 h induction, cells were harvested and resuspended in 1/5 the original volume of fresh LB medium containing kanamycin (25 µg/mL). Flavonoid substrate (50 μM) was added to a fresh culture followed by incubation at 25 °C. An aliquot of the culture was harvested at a selected time point and centrifuged to obtain cell-free medium. The medium was extracted twice with ethyl acetate to recover the reactants and products, and the resulting extracts were evaporated to dryness. Residues were redissolved in a small volume of methanol, and analyzed by HPLC using the elution conditions described above.

### 3.6. Identification of Methylated Products

The methylation products of flavonoid substrates were identified using ^1^H, ^13^C and HMBC NMR spectroscopy. NMR spectra were recorded in CD_3_OD at room temperature using an Avance NEO 400 NMR spectrometer (Bruker, Rheinstetten, Germany). Tetramethylsilane was used as an internal standard. Chemical shifts are given on the δ-scale.

## 4. Conclusions

Biosynthetic enzymes have been considered as useful tools for the biotechnological production of valuable natural products. In the present study, a putative flavonoid OMT, PfOMT3, was isolated from perilla leaves. To characterize its biochemical properties, PfOMT3 was expressed in *E. coli* and affinity purified. An OMT activity assay of recombinant PfOMT3 showed that PfOMT3 is an FOMT, which methylates diverse flavonoids regardless of their backbone structure. PfOMT3 regiospecifically transfers a methyl group to the 7-OH of flavonoids. Kinetic studies revealed that chrysin, naringenin and apigenin are preferred substrates of PfOMT3. Engineered *E. coli* bearing PfOMT3 successfully bioconverted naringenin and kaempferol to the 7-*O*-methylated products sakuranetin and rhamnocitrin, respectively. These findings suggest that PfOMT3 can be a useful tool for the biotechnological production of 7-methoxyflavonoids.

## Figures and Tables

**Table 1:** Substrate preference of recombinant *Perilla frutescens O*-methyltransferase3 (PfOMT3).

Substrate	Relative Activity (%) ^a^
*Flavanone*	
Naringenin	97.79
Eriodictyol	53.13
Taxifolin	ND ^b^
*Flavone*	
Apigenin	95.17
Luteolin	46.80
Chrysin	100.00
4′,7-Dihydroxyflavone	62.76
*Flavonol*	
3,6-Dihydroxyflavone	ND ^b^
Kaempferol	40.34
Quercetin	ND ^b^

^a^ To determine relative activity, 50 µM of each substrate, 100 µM SAM and 50 µg of recombinant PfOMT3 were combined in 20 mM Tris-HCl buffer (pH 7.5). ^b^ ND indicates that 7-*O*-methylated products were not detected in the PfOMT3 reaction mixture.

**Table 2:** Kinetic parameters of recombinant PfOMT3 ^a^.

Substrate	*K*_M_ (μM)	*V*_max_ (nmol min^−1^ mg^−1^)	*k*_cat_ (min^−1^)	*k*_cat_/*K*_M_ (M^−1^ s^−1^)
Naringenin	13.40 ± 1.29	51.65 ± 1.21	2.116	2.632 × 10^3^
Eriodictyol	13.08 ± 1.42	7.84 ± 0.513	0.321	4.094 × 10^2^
Chrysin	1.31 ± 0.25	2.05 ± 0.22	0.084	1.067 × 10^3^
Apigenin	3.72 ± 0.05	13.32 ± 1.12	0.546	2.442 × 10^3^
Luteolin	34.21 ± 4.65	4.18 ± 0.16	0.171	8.335 × 10^1^
Kaempferol	17.88 ± 0.49	5.81 ± 0.26	0.238	2.219 × 10^2^

^a^ Results are means ± SD of triplicated experiments.

## Supplementary Materials

Supplementary Materials are available online. Figure S1: Nucleotide and amino acid sequences of PfOMT3. Figure S2: HPLC analysis of PfOMT3-catalyzed reactions with flavonoid substrates. Figure S3: HPLC analysis of PfOMT3-catalyzed reactions with phenylpropanoid substrates. Table S1: ^1^H (400 MHz) and ^13^C (100 MHz) NMR chemical shifts of the methylated products of naringenin and kaempferol by PfOMT3 reaction (in CD_3_OD, chemical shifts in ppm).
